# Spatial transcriptomics in cancer research and potential clinical impact: a narrative review

**DOI:** 10.1007/s00432-024-05816-0

**Published:** 2024-06-08

**Authors:** Michael A. Cilento, Christopher J. Sweeney, Lisa M. Butler

**Affiliations:** 1 South Australian Immunogenomics Cancer Institute, The University of Adelaide, Adelaide, SA Australia; 2 South Australian Health and Medical Research Institute, Adelaide, SA Australia; 3 The Queen Elizabeth Hospital, Woodville South, SA Australia

**Keywords:** Precision oncology, Genetics, Spatial transcriptomics, Tumor microenvironment, Solid tumors

## Abstract

Spatial transcriptomics (ST) provides novel insights into the tumor microenvironment (TME). ST allows the quantification and illustration of gene expression profiles in the spatial context of tissues, including both the cancer cells and the microenvironment in which they are found. In cancer research, ST has already provided novel insights into cancer metastasis, prognosis, and immunotherapy responsiveness. The clinical precision oncology application of next-generation sequencing (NGS) and RNA profiling of tumors relies on bulk methods that lack spatial context. The ability to preserve spatial information is now possible, as it allows us to capture tumor heterogeneity and multifocality. In this narrative review, we summarize precision oncology, discuss tumor sequencing in the clinic, and review the available ST research methods, including seqFISH, MERFISH (Vizgen), CosMx SMI (NanoString), Xenium (10x), Visium (10x), Stereo-seq (STOmics), and GeoMx DSP (NanoString). We then review the current ST literature with a focus on solid tumors organized by tumor type. Finally, we conclude by addressing an important question: how will spatial transcriptomics ultimately help patients with cancer?

## Introduction

Spatial transcriptomics (ST) is the study and quantification of messenger RNA (mRNA) transcripts as a surrogate for gene expression in the spatial context of cancer and the associated microenvironment (Marx 2021; Moses and Pachter 2022). The use of ST in cancer research has the potential to have a significant impact on patients through its application in precision oncology care. This highly detailed illustration of gene expression has been made possible with the recent development of a number of spatial platforms.

This review summarizes tumor sequencing in the clinical setting and tumor sequencing in the research laboratory, including bulk and spatial methods. We then review some of the current key ST literature categorized by tumor type, with an emphasis on novel studies that directly analyzed original human samples. We conclude with a discussion on the future directions for the integration of ST into the clinical care of patients with cancer.

## Tumor sequencing in the clinic

### Precision oncology

Precision medicine, sometimes called personalized medicine, refers to the selection of specific therapeutics for a patient based upon the unique characteristics of that individual or the disease being treated (Jain 2002). Precision oncology is the application of this concept to cancer treatment and most commonly refers to the use of molecular tumor profiling to guide the selection of cancer therapies (Yates et al. 2018). This approach to cancer therapy has led to the rapid development of targeted therapies for most solid tumors, many of which now define the standard-of-care options. Examples include cancers such as malignant melanoma with *BRAF* variants, ovarian carcinomas with *BRCA* alterations and non-small cell lung carcinoma, where the list of targeted treatments is ever expanding but already includes *EGFR*, *ALK*, *ROS-1*, *NTRK*, *MET*, *RET*, *BRAF*, and *KRAS* (Imyanitov et al. 2021).

The advent of precision oncology has largely come about due to the increasing accessibility of comprehensive genomic profiling (CGP), which in turn has driven drug development. However, significant disparities remain globally for patients with cancer both in access to testing and affordability of targeted treatments (Mateo et al. 2022). The CGP can help guide decision-making in solid tumor management; however, prior to testing, there are many practical factors for clinicians to consider.

### Tumor tissue processing

Genomic sequencing of cancer starts with the critical initial step of obtaining suitable tumor tissue for testing. Often clinicians will rely on tumor tissue that has already been collected previously during the patient’s treatment journey, so-called ‘archival’ tissue. Archival tissue can be obtained from formalin-fixed paraffin-embedded (FFPE) tumor blocks or unstained tissue on microscope slides prepared at the time of previous surgical resection or from previous tumor biopsies. The reliance upon FFPE tissue comes with several limitations, including degraded nucleic acids, mutational noise, and artifacts from fixation (Do and Dobrovic 2015). Many of these limitations can be overcome by obtaining ‘fresh’ biopsies. However, laboratory advances are continually being made to optimize the extraction of DNA from FFPE tissue (Inoue et al. 2021; Oba et al. 2022), as most clinical tissue samples are stored in this way.

Next, performing next-generation sequencing (NGS) of tumors requires tissues to be homogenized prior to sequencing; therefore, the result is an average read-out of the genomic material present, which may include both benign and malignant areas depending on the sample. This also results in the loss of any specific spatial tumor context.

### Variants and the Molecular Tumor Board (MTB)

Finding variants in the context of clinical cancer CGP involves identifying genetic changes, such as mutations and alterations in a tumor’s DNA. The first step after sequencing the DNA involves ‘sequence alignment,’ whereby the DNA obtained from the tumor sample is aligned to the reference genome. Variant calling algorithms are subsequently used to detect differences in the form of mutations or other alterations (Feng et al. 2023). The next key step is the laboratory-clinic interface, where the detected variants are clinically interpreted to inform treatment decisions. This process is often carried out by a Molecular Tumor Board (MTB).

## Tumor sequencing in the research laboratory

### Cancer genomics and transcriptomics

Genomics and transcriptomics both play key roles in cancer research. Briefly, genomics refers to the entire set of genes in a tumor determined by analyzing DNA to identify variants such as mutations, deletions, or amplifications that may cause cancer or predict treatment benefit. On the other hand, transcriptomics focuses on the RNA transcripts produced by genes, providing an indication of the dynamic pattern of gene expression at a given timepoint.

### Bulk methods

**Fig. 1:**
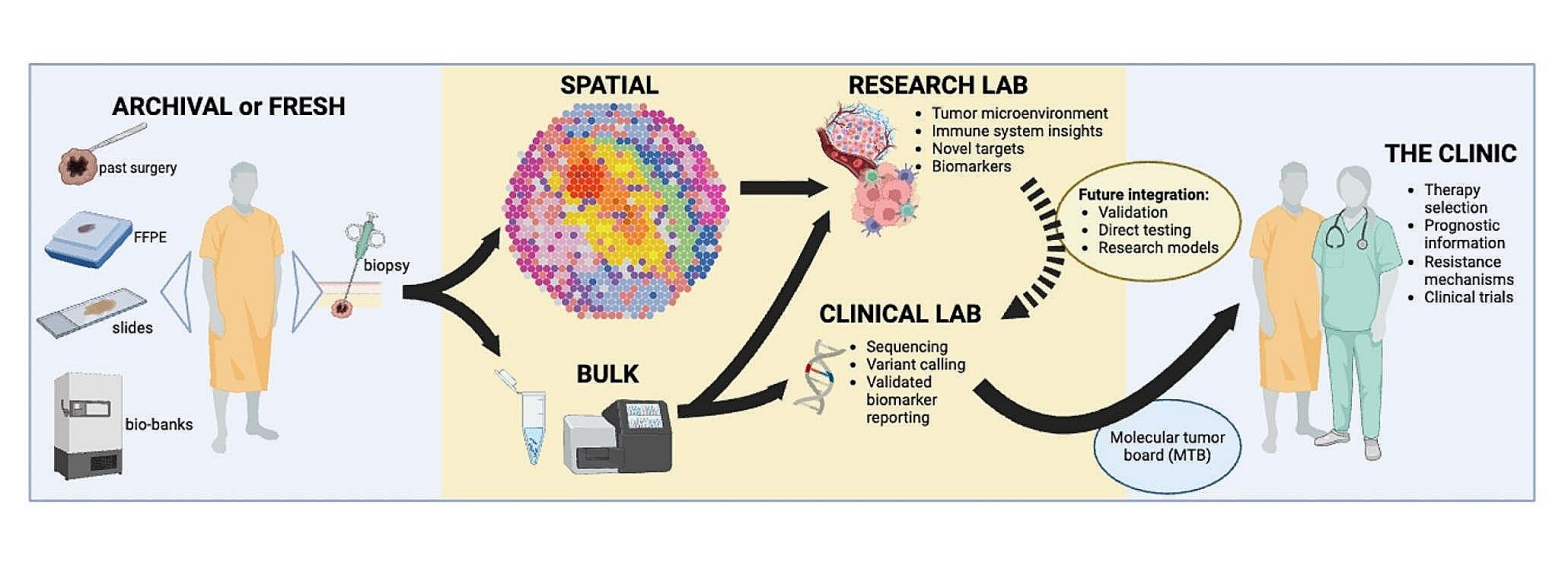
Complimentary bulk and spatial methods have the potential to accelerate the discovery and clinical translation of research insights from testing tumor tissue

Transcriptomics utilizes various techniques to study the transcriptome, that is, the quantification of all RNA transcripts, which can be used as a surrogate for gene expression. The understanding of genomic function has increased rapidly with the widespread use of RNA-seq in molecular biology (Stark et al. 2019). Like in genomic analysis, the main limitation of this bulk transcriptomic approach is the loss of a cell type-specific understanding and spatial context of gene expression, both of which are now being explored through single-cell sequencing and ST. The complimentary nature of bulk and spatial approaches may allow integration of both methods in clinical and research settings in the near future (Fig. 1).

### Single-cell sequencing

Single-cell RNA sequencing (scRNA-seq) provides insights into the heterogeneity of tissue samples and cell populations. Briefly, isolation of individual cells often involves cell sorting or microfluidic techniques. The genetic content of each cell is subsequently bound to a specific detection probe functioning as a ‘barcode’. The barcoded fragments for hundreds to tens of thousands of cells are pooled, extracted, and subsequently sequenced. Although scRNA-seq is a powerful stand-alone tool for investigating heterogeneity, it still has the limitation of tissue dissociation and therefore loss of spatial context. This issue has now been addressed with a variety of spatial methods, including ST.

## Spatial transcriptomics (ST)

### Overview

Spatial transcriptomics (ST) is the study and quantification of messenger RNA (mRNA) transcripts as a surrogate for gene expression in the spatial context of cancer cells and their associated microenvironment (Marx 2021; Moses and Pachter 2022). Multiple ST technologies have been developed, all of which result in the generation of large volumes of sequencing data for each specimen that is analyzed. Recently, advances in technology have dramatically improved the speed and quality of data acquisition and processing, which has given rise to extremely detailed resolution. Several reviews of the investigative methods employed for ST have been published in the literature in recent years (Asp et al. 2020; Liao et al. 2021; Moses and Pachter 2022; Rao et al. 2021). While the catalog of proprietary systems available for spatial transcriptomic analysis is rapidly expanding, the methods employed are generally categorized by the way in which the data are obtained and can be separated broadly into imaging-based methods and sequencing-based methods (Table 1).

**Table 1:** Overview of spatial transcriptomic (ST) technologies commonly applied to cancer research. FFPE: formalin-fixed paraffin embedded; FF: fresh frozen; c-t-c: center-to-center distance; *denotes the capture area is less than the size of a single cell (although this is not considered equivalent to dedicated scRNA-seq techniques)

Method	Product (Company)	Single-cell resolution*	Resolution	Tissue capture area	Tissue types	Number of human genes	Protein capability	Estimated workflow time	Notable advantages or disadvantages
**Imaging-based**									
In Situ Hybridization (ISH)	MERFISH (Vizgen)	Yes	0.1 μm	10 × 10 mm	FFPE, FF	100 − 10,000 genes	Co-detection possible on same sample	36 h	Advantage: error-robust binary barcoding
	CosMx Spatial Molecular Imager (NanoString)	Yes	0.05 μm	20 × 15 mm	FFPE, FF	1,000 RNA transcripts	CosMx Protein Assay (up to 68 proteins)	3 days minimum	Disadvantage: long imaging time
In Situ Sequencing (ISS)	Xenium (10x Genomics)	Yes	0.05 μm	12 × 24 mm	FFPE, FF	266–480 genes on panel	Protein panels not yet available (announced as a ‘future product’)	2 days	Disadvantage: panel-based assay currently limits number of genes
**Sequencing-based**									
	Spatial Transcriptomics (ST)	No	100 μm spot, 200 μm c-t-c distance	(now commercialized as Visium)	FFPE, FF	Whole transcriptome	No	2 days	Disadvantage: low resolution
	Visium spatial gene expression (10x Genomics)	No (captures between 1–10 cells per spot)	55 μm spot, 100 μm c-t-c distance	6.5 × 6.5 mm	FFPE, FF	Whole transcriptome	NGS-read out of Protein Expression data.	1 day	Disadvantage: resolution has been superseded by newer technology
	Visium HD (10x Genomics)	Yes	2 × 2 μm squares, with no gaps	6.5 × 6.5 mm (two capture areas per slide)	FFPE, FF	Whole transcriptome	NGS-read out of Protein Expression data.	2–3 days (8 h hands-on time)	Advantage: high resolution whole transcriptome
	Stereo-seq (STOmics)	Yes	0.2 μm spot, 0.5 μm c-t-c distance	10 × 10 mm mounted on glass slide, larger chips up to 13 × 13 cm available	FF	Whole transcriptome	Multiplexed immunofluorescence (mIF) co-detection on 1 × 1 cm chip only	1–2 days	Advantage: currently has largest available slide/chip
	GeoMx Digital Spatial Profiler (NanoString)	No	10–600 μm are of interest selection	35.3 × 14.1 mm	FFPE, FF	18,000 RNA transcripts	570 + protein targets available	1 day	Advantage: multiple samples can be placed on slide, not limited by capture areas

### Imaging-based methods: ISH and ISS

Imaging-based methods can be broadly subdivided into in situ hybridization (ISH) or in situ sequencing (ISS) methods.

#### In situ hybridization (ISH)

Single-molecule FISH (smFISH) is a quantitative method that uses five fluorophores per DNA or RNA molecule to image probe-labeled transcripts. However, this technique is limited by the number of detectable genes due to spectral overlap (Lewis et al. 2021). This issue has been addressed and overcome by employing multiple rounds of sequential hybridization, such as sequential fluorescence in situ hybridization (seqFISH) using color barcodes (Williams et al. 2022) or multiplexed error-robust FISH (MERFISH), which employs binary barcodes (Chen et al. 2015). While barcoding and sequential hybridization have allowed scaling and multiplexing of FISH techniques, they are limited by the increased time required for imaging, as well as the relatively small area of tissue that can be imaged.

The *CosMx Spatial Molecular Imager (SMI)* (NanoString) resolves RNA and protein expression at single-molecule resolution. The technology involves tissue permeabilization, probe hybridization, and slide assembly insertion in the *SMI*. Fluorescently labeled secondary probes containing a UV cleavable linker are then added. *CosMx* allows simultaneous imaging and quantification of 1000 + RNA and 64 + protein targets at subcellular resolution (He et al. 2022).

#### In situ sequencing (ISS)

In situ sequencing (ISS) is considered an imaging-based technique, as the method also involves visualization of mRNA directly in a section of tissue or cell sample. ISS enables in situ targeted gene expression profiling, a method commercialized as *Cartana* and subsequently acquired by 10x Genomics (Lewis et al. 2021). In this method padlock probes are hybridized to transcripts, probe ends are ligated, and the products are amplified using rolling circle amplification (RCA). Next, fluorescent probes are hybridized to the RCA product (Williams et al. 2022). The resulting fluorescent DNAs are read using iterative imaging technologies. This modification of *Cartana* technology was subsequently launched as *Xenium* (10x Genomics) in December 2022. *Xenium* is a panel-based assay that can currently be run with a maximum panel of 480 gene markers, although significantly larger panels are expected with recent announcements of plans for a 5 K (5000 gene) panel.

### Sequencing-based methods

#### Microdissected specimens

Spatial regions of interest (ROIs) can also be isolated manually from tissues using microdissection, for example, to isolate a tumor deposit from an immune cell infiltrate. Laser capture microdissection (LCM) is the most common physical microdissection technique in which selected areas are dissected using a UV laser or by fusion of tissue with a membrane via an IR laser. These selected regions are then subjected to RNA extraction.Gene expression profiling is performed with a complementary DNA (cDNA) microarray or RNA sequencing (RNA-seq) or by dissociation into single cells for single-cell RNA-seq (scRNA-seq)(Moses and Pachter 2022). While this approach can yield detailed results for the selected ROIs, the major limitation is the impracticality of dissecting multiple discrete tissue compartments across a larger tissue sample(Asp et al. 2020).

#### NGS with spatial barcoding

The spatial location of transcripts can also be determined by capturing them directly from tissue sections(Moses and Pachter 2022). The samples are positioned on arrayed reverse transcription primers with unique positional barcodes via a strategy first described as “spatial transcriptomics” (*ST*). The mRNA captured with these spatially barcoded probes is converted to cDNA and then sequenced using previously established next-generation sequencing (NGS) platforms.

The resolution of such an approach is dependent upon the size of each capture spot. The initial *ST* platform had a spot diameter of 100 μm with a center-to-center distance of 200 μm(Ståhl et al. 2016). The product later commercialized by 10x Genomics, called *Visium*, increased the resolution by decreasing the size of each capture spot to 55 μm in a hexagonal array, in which each capture spot can contain approximately 1 to 10 cells depending on the tissue analyzed. In the latest iteration, *Visium HD* further increased the resolution with each slide featuring 2 × 2 μm barcoded squares, with no gaps between squares. Additional barcoded products, such as *Slide-seq* and *Slide-seqV2*, have a 10 μm diameter resolution (Stickels et al. 2021). Recently, the *Stereo-seq* (STOmics) platform has refined the microarray pattern to a 200 nm (0.2 μm) spot resolution (Chen et al. 2022). Traditionally, analysis of transcriptomes has been dependent upon the use of fresh frozen tissue to preserve mRNA and prevent degradation. However, newer technologies can now employ additional steps to allow formalin-fixed paraffin-embedded (FFPE) tissues to be sequenced, an approach compatible with many clinical specimens that in turn creates great opportunity for translational research.

Another commercially available sequencing-based ST product is the *GeoMx Digital Spatial Profiler (DSP)* by NanoString, which combines immunofluorescence techniques with optical barcoding technology (Li and Wang 2021). The platform uses gene-specific probes linked to unique barcodes for spatial mRNA analysis on tissue slides. After staining with fluorescent antibodies, regions of interest (ROIs) are selected for barcode collection, library construction, and sequencing. It accommodates more samples per slide than array-based platforms and allows for customization with custom probes. Challenges include validating antibody staining for satisfactory results (Y. Wang, B. Liu et al. 2023a).

Regardless of the specific platform used, analysis of data obtained via spatially barcoded approaches requires computational reconstruction. Given that each barcoded capture spot may contain overlapping cells, the mRNA detected will include content from more than one cell type. Therefore, a process of deconvolution must be performed to infer what cell types are present (Williams et al. 2022). Deconvolution can be achieved either through utilizing publicly available data or preferably via generation of a concordant single-cell RNA sequencing dataset from the same tissue samples undergoing spatial analysis (Kleino et al. 2022; Mañanes et al. 2024).

## Spatial transcriptomics in solid tumors: what we’ve learned thus far

**Fig. 2:**
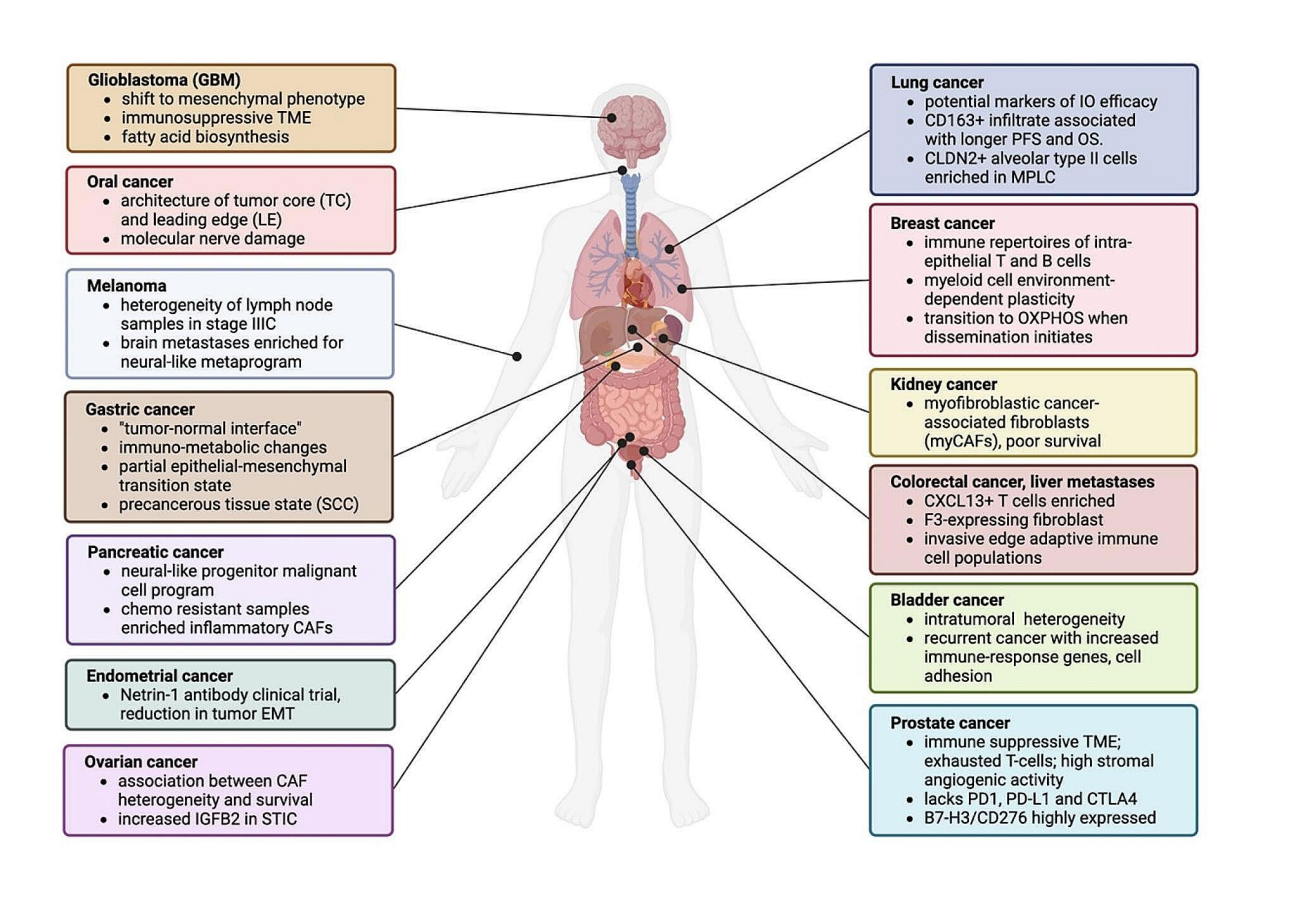
Overview of spatial transcriptomic insights into solid tumors

There has recently been an explosion in the use of ST for cancer research, with numerous publications across multiple cancer types. Almost all solid organ malignancies are represented in the literature. Here, we review the current key literature categorized by tumor type (Fig. 2), with an emphasis on novel studies that directly analyzed original human samples, excluding papers where the approach was purely an *in silico* (computational) investigation of previously available datasets (See Table 2).

**Table 2:** Cancer-specific research using spatial transcriptomics (ST)

Tumor type	ST method	ST sample size	Novel spatial insights	Ref.
Brain				
GBM	‘Spatial transcriptomics’	12 samples	IL-10-releasing HMOX1(+) myeloid cells (**located in mesenchymal-like tumor regions**) drive T-cell exhaustion, contributing to **immunosuppressive TME**. Additionally, single-cell data generated for 4 patients (1 IDH-mutated glioma, 3 IDH-WT GBM) in addition to the ST experiment.	(Ravi et al. 2022a)
GBM	Visium	9 samples	Determined the **cellular composition and transcriptional states** in primary and recurrent glioma and identified three compositional spatial ‘tissue-states’. Fatty acid biosynthesis was enriched in specific tissue-states and was associated with recurrent GBM and **shorter survival**.	(Al-Dalahmah et al. 2023)
GBM	Visium	28 specimens	Integrated **metabolomics** (*n* = 6) and **proteomics** (*n* = 6) on adjacent tissue sections to show locoregional **tumor-host interdependence**, inferred copy-number alterations to show “**spatially cohesive organization** of subclones” associated with reactive transcriptional programs.	(Ravi et al. 2022b)
GBM	Visium	24 patients	Assessed patient samples based on status as newly diagnosed (*n* = 7), recurrent (*n* = 5), or neoadjuvant pembrolizumab and anlotinib (*n* = 12). **Tumor core** showed increased gene signatures for hypoxia, angiogenesis, inflammation **compared to paratumor**. **TAM subpopulations** highly expressing *SIGLEC9* gene preferentially accumulated in **non-responders to anti-PD1**.	(Mei et al. 2023)
GBM	Visium	5 patients	Titled “Myeloid-specific KDM6B inhibition sensitizes glioblastoma to PD1 blockade” this study identified **high expression of the epigenetic enzyme KDM6B** in **intratumoral** immune-suppressive myeloid cell subsets. Mouse models used to show potential as a therapeutic target to improve responsiveness to immunotherapy.	(Goswami et al. 2023)
GBM	GeoMx DSP	6 FFPE samples, 12 ROIs/sample	GBM characterized by a **shift to mesenchymal phenotype**, mediated by activator protein 1. CD8 + T-cells in primary tumors were sparse, isolated and frequently **confined to the perivascular space.** Increased T-cell abundance at recurrence was prognostic and correlated with hypermutation status.	(Wang et al. 2022a)
Head and neck				
Oral cancer	Visium	12 samples fresh-frozen	Characterized the transcriptional architecture and differences between malignant cells in the **tumor core (TC)** and **leading edge (LE)**. Demonstrated the gene expression profile associated with the LE is conserved across different cancers.	(Arora et al. 2023)
Oral cancer	Visium	5 specimens	Pairwise biopsies collected from 9 individuals to investigate stepwise progression from precancerous to cancerous. **VEGFA and TGFβ signaling** were found to be enriched in **oral SCC initiation**. The mesenchymal CAF subcluster dominated cancer initiation and was **spatially closer to the epithelium** when compared to inflammatory CAFs.	(Sun et al. 2023b)
Oral cancer	GeoMx DSP	8 samples	Investigated **perineural invasion** (PNI) to show nerves near tumor have unique transcriptomic signatures, suggesting **nerve damage is molecular** rather than physical.	(Schmitd et al. 2022)
Breast				
Breast cancer	Laser capture microdissection	7 samples	Applied laser capture microdissection, followed by sequencing. Found immune repertoires of **intra-epithelial T and B cells** were consistently less diverse and more clonal than those in stroma.	(Romanens et al. 2023a)
Breast cancer	‘Spatial transcriptomics’	1 sample	Original 2016 publication in *Science*, demonstrated ST method with arrayed oligonucleotides with positional barcordes. ST applied to mouse brain and human breast cancer tissue containing DCIS and invasive components. **Invasive component** revealed **high expression of ECM–associated genes**.	(Ståhl et al. 2016)
Breast cancer HER2	‘Spatial transcriptomics’	8 patients (36 sections)	Demonstrated T cell and myeloid interaction and **co-localization**. Showed a certain **spatial heterogeneity of *ERBB2*expression**. From the 8 individual patients, a total of 36 sections were analyzed.	(Andersson et al. 2021)
Breast cancer	Visium	4 original specimens	Breast cancer samples including ER + and triple-negative tumors used to provide a **comprehensive transcriptional atlas** of the **cellular architecture** of breast cancer. In addition to the 4 original specimens, analysis also included 2 specimens from another lab.	(Wu et al. 2021)
Breast cancer	Visium	8 patients	Analyses on tumor and adjacent normal tissue as well as axillary lymph nodes and peripheral blood mononuclear cells (PBMCs). Found **myeloid cells** exhibited **environment-dependent plasticity**.	(Mao et al. 2023)
Breast TNBC	Visium	28 sections from 14 patients	Focus on defining tumor architecture of TNBC. Found race-associated differences in **hypoxic tumor content** and regions of **immune-rich infiltrate**.	(Bassiouni et al. 2023)
Breast	Visium	4 patients	Metabolism focus, detected a transition between glycolysis and **oxidative phosphorylation** (OXPHOS) when **dissemination initiates**. The distinct cluster is **distributed along the tumor leading edge**.	(Liu et al. 2023b)
Thoracic				
Lung cancer	Visium	4 patients (12 samples)	Population of epithelial cells, termed CLDN2 + alveolar type II cells, shown to be enriched in **multiple primary lung cancers (MPLC)**. However, the **spatial architecture of MPLC remained unclear**, with differences seen between separate tumor lesions in individual patients.	(Wang et al. 2023b)
Lung cancer	GeoMx DSP	8 samples	Found a relatively higher frequency of predicted neoantigens located within HLA-I presentation hotspots in CD3 + CD8 + **T cell-excluded tumors based on ST and spatial proteomics**.	(Kraemer et al. 2023)
Lung cancer	GeoMx DSP	Tissue microarray (TMA)	Utilized a TMA and analyzed 33 tumor ROIs, 23 stroma ROI. Identified several **potential markers of immunotherapy efficacy** including IL2, CD25, CD44 and SPP1. Demonstrated that **stromal IL2 mRNA levels** positively correlated with the expression of pro-apoptotic markers cleaved caspase 9.	(Monkman et al. 2023)
Lung cancer	GeoMx DSP	16 patients	Checkpoint inhibitor treated patients assessed, showed **low intertumoral CD163 + cell infiltration** was associated with longer progression-free survival and overall survival. ST profiles showed upregulation of ITGAM, CD27, and CCL5 in tumors with high CD163+.	(Larroquette et al. 2022)
Lung cancer	GeoMx DSP	44 patients	Lung cancer **brain metastasis** specimens analyzed, **T-cell- and antibody-mediated adaptive immune responses** specifically compromised.	(Zhang et al. 2022)
Upper gastrointestinal				
Gastric cancer	Visium	7 samples	ST integrated with mass spectrometry imaging (MSI) showing metabolic reprogramming. Immune cell-dominated **tumor-normal interface** with significant immunometabolic alterations.	(Sun et al. 2023a)
Gastric cancer	Visium	4 samples	Demonstrated the presence of GC cells in a partial **epithelial–mesenchymal transition state**. Mouse models used to show TGFβ drives the switch from normal to mesenchymal stem cell state. ST showed coexpression of epithelial and mesenchymal genes **within adjacent lesions** of a single primary gastric cancer.	(Jang et al. 2023)
Pancreatic cancer	Visium	31 patients	Chemotherapy resistant samples **enrichment of inflammatory cancer-associated fibroblasts in the TME** that upregulate metallothioneins.	(Cui Zhou et al. 2022)
Pancreatic cancer	Visium	8 samples	Substantiate the **exhausted phenotype of CD8 T cells** and **immunosuppressive features** of myeloid cells, **comparing tumor with adjacent normal tissue**.	(Yousuf et al. 2023)
Esophageal SCC	GeoMx DSP	19 samples	Assessed **precancerous and cancerous tissues**, showed expression of TAGLN2 increases, while CRNN expression level decreases along the continuum of progression.	(Liu et al. 2023a)
Pancreatic cancer	GeoMx DSP	21 specimens	Neoadjuvant therapy seemed to elicit an enriched **neural-like progenitor (NRP) malignant cell program**, compared to untreated patient specimens. Colocalization of the NRP malignant program, neurotropic CAF program and CD8 + T cells in **one multicellular community** suggested an interplay among these cell types.	(Hwang et al. 2022)
Lower gastrointestinal				
CRC	Visium	4 patients	Interaction of ***FAP*** **+ fibroblasts** and ***SPP1*** **+ macrophages** in colorectal cancer, authors propose stimulate the formation of **immune-excluded desmoplastic structure** and limit the T cell infiltration.	(Qi et al. 2022)
CRC liver metastases	Visium	6 patients, 27 samples	Charts the cellular landscape of CRC and liver metastatic tumors. CXCL13 + **T cells** are enriched in liver metastatic tumors. F3-expressing **fibroblast** subset enriched in the primary tumors. Compared with paratumor tissues, tumor tissues were enriched with cell cycle–related pathways. They also showed decreased ratio of plasma cells.	(Wang et al. 2023a)
CRC liver metastases	Visium	4 patients	Reports this discovery of two distinct **senescent metastatic cancer cell subtypes**, transcriptionally located at the opposite pole of epithelial to mesenchymal transition.	(Garbarino et al. 2023)
CRC + oral SCC	Visium and GeoMx DSP	2 samples	Intratumoral bacteria tend to **populate niches that are less vascular and highly immunosuppressed**.	(Galeano Niño et al. 2022)
CRC liver metastases	GeoMx	4 matched primary CRC and liver metastases	ST demonstrated that the **invasive edge** of the metastases of long-term survivors was characterized by **adaptive immune cell populations** enriched for type II IFN signaling and MHC-class II antigen presentation.	(Wood et al. 2023)
Urological				
Prostate cancer	‘Spatial transcriptomics’	12 specimens	Focus on illustrating heterogeneity. Distinct expression profiles shown for the different tissue components, such as **stroma, normal and PIN glands, immune cells, and cancer**.	(Berglund et al. 2018)
Prostate cancer	Slide-seqV2	4 samples, 2 normal and 2 tumors	Localized prostate cancer samples assessed with ST, illustrated **immune suppressed microenvironment** by the presence of suppressive myeloid populations, exhausted T cells, and high stromal angiogenic activity.	(Hirz et al. 2023)
Bladder cancer	Visium	4 tumors	Primary and recurrent samples compared. **Recurrent group** demonstrating higher levels of heterogeneity, increased expression of **immune-response genes** and **cell adhesion.**	(Lindskrog et al. 2023)
Bladder cancer	Visium	6 tissues	Recurrent tumors show increase in activity between **cancer-associated fibroblasts (CAFs)** and **malignant cells**, as compared to primary tumors. Noted that CD8 + T cells and plasmacytes were **located proximal to the tumor boundary** in recurrent tumors.	(Shi et al. 2023)
Renal cell carcinoma	Visium	2 samples	Correlation between mesenchymal-like ccRCC cells and **myofibroblastic cancer-associated fibroblasts (myCAFs**), which are both enriched in metastases and correlate with poor patient survival.	(Davidson et al. 2023)
Renal cell carcinoma	Visium	6 patients, 10 samples	Mechanisms explaining lack of response to immunotherapy in high-risk patients, including **exhausted immune cells** and lack of expression of *PD-1*, *PD-L1* and *CTLA4* genes.	(Raghubar et al. 2023)
Prostate cancer	GeoMx DSP	Tissue microarray (TMA)	TMA constructed from 27 patients, in total, 168 tumor cores from 56 tumors used. Metastatic prostate cancer **immune infiltrate lacks PD1, PD-L1 and CTLA4**. However, the **B7-H3/CD276 highly expressed**.	(Brady et al. 2021)
Gynecological				
Ovarian cancer	‘Spatial transcriptomics’	4 samples	Association between **cancer-associated fibroblasts (CAF) heterogeneity** and long-term survival in patients with advanced HGSC. ST used to depict ligand–receptor cross-talk **heterogeneity at the tumor-stroma interface**.	(Ferri-Borgogno et al. 2023)
Endometrial cancer	Visium	4 specimens	Netrin-1 antibody **clinical trial** with novel integration of ST. Net **reduction in tumor EMT**, associated changes **in immune infiltrate** and increased interactions between cancer cells and the TME.	(Cassier et al. 2023)
Ovarian cancer	GeoMx DSP	16 samples	Focus on ovarian cancer precursors (STIC). Increased levels of **insulin-like growth factor binding protein-2** (IGFBP2) in STIC (but **not in surrounding normal fallopian tube epithelia**) as a mechanism of proliferation.	(Wang et al. 2023b)
Skin				
Cutaneous SCC	Visium	10 samples	Tumor **leading edge illustrated**, increased CAFs and endothelial transcripts described. Increased expression of **basal tumor genes** identified.	(Ji et al. 2020)
Melanoma brain metastases	Slide-seqV2	16 tissue sections	Showed cancer cells in brain **metastases enrich for a neuronal-like metaprogram**. **Macrophages** shown to have a **pro-tumorigenic phenotype** in brain metastases. Authors showed **spatially dichotomous expression** of oxidative phosphorylation and glycolysis metabolic pathways in cancer cells.	(Biermann et al. 2022)
Melanoma	‘Spatial transcriptomics’	4 samples	Early publication from 2018, examined 4 lymph node samples from patients with stage IIIC, described **heterogeneity of gene expression** in samples.	(Thrane et al. 2018)

### Brain: Glioblastoma

Primary brain tumors, such as glioblastoma (GBM), are known to be among the most treatment-resistant solid tumors. Various spatial profiling methods have been applied in brain tumor research to investigate this highly complex, treatment-resistant TME, the early applications of which have previously been reviewed (Kalita-de Croft et al. 2021).

More recently, spatial research into the lymphoid cell population of GBM patients revealed that T-cell dysfunction was associated with an increased response to interleukin 10 (IL-10), which was spatially related to the presence of *HMOX1 +* myeloid cells (Ravi et al. 2022). This provided novel insights into the immune-suppressed TME of GBM. The role of immune-suppressive myeloid cells, which exhibit high expression of the epigenetic enzyme KDM6B, has also been recently reported (Goswami et al. 2023).

The integration of metabolomics and ST data revealed an association between reactive hypoxia programs and specific metabolic alterations in some regions, including enrichment of genes related to phosphoadenylate metabolism (Ravi et al. 2022b). Comparative analysis of matched primary and recurrence samples has shown that patients with GBM exhibit a shift in proneural (PN) to mesenchymal (MES) phenotypes at recurrence (Wang et al. 2022a). Additionally, matched primary-recurrence samples have also been utilized to reveal distinct tissue states; specifically, fatty acid biosynthesis enrichment in “the tissue state defined by the cohabitation of astrocyte-like/mesenchymal glioma cells, reactive astrocytes, and macrophages” is associated with recurrent GBM and shorter survival (Al-Dalahmah et al. 2023). Patient cohorts receiving neoadjuvant immunotherapy have also been analyzed, demonstrating the role of tumor-associated macrophage (TAM) subpopulations, particularly the presence of *SIGLEC9 +* TAMs, in immunotherapy nonresponders (Mei et al. 2023).

### Head and neck cancer

Insights into the architecture of the TME are now possible with ST. Oral squamous cell carcinoma (SCC) researchers have identified unique transcriptional signatures of the cell layer at the border of the tumor (termed the leading edge) as distinct from the tumor core; additionally, unique ligand–receptor interactions have also been identified at the leading edge (Arora et al. 2023).

The model of oral SCC initiation from a precancerous lesion is well known; however, the underlying mechanisms have only recently been probed using scRNA-seq and ST. VEGFA and TGFβ signaling were found to be enriched in oral SCC initiation based on analysis of paired samples across the spectrum of normal, dysplastic, and cancerous tissues (Sun et al. 2023b).

The presence of perineural invasion (PNI) in oral SCC is a known factor increasing the risk of poor clinical outcomes. Investigators applied *GeoMx* DSP to eight tumor samples with PNI to show that nerves in close proximity to tumors have unique transcriptomic signatures, including changes to myelin reflecting injury and enrichment of axonogenesis and stress response genes, the gradients of which are dependent on nerve-tumor distance (Schmitd et al. 2022). The investigators proposed that nerve injury is molecular rather than physical based upon these findings.

### Breast cancer

The original description of the “spatial transcriptomics” method involved analysis of the mouse brain and a human breast cancer specimen (Ståhl et al. 2016), and since this time, the use of ST in breast cancer research has accelerated rapidly. Initially, researchers focused their work on descriptive studies providing a transcriptional atlas of the cellular landscape of estrogen receptor (ER)-positive and triple-negative breast cancer (Wu et al. 2021), as well as HER2-positive breast cancer (Andersson et al. 2021). However, recently, more specific lines of investigation have been pursued.

The immune microenvironment of breast cancer has been a research area of great interest. Mao and colleagues performed an integrated analysis of patient blood, lymph node, tumor and adjacent normal tissue to illustrate the environment-dependent changes in multiple cell types. They showed that axillary lymph nodes transform into a tumor-like state with T-cell exhaustion, as well as a decrease in B cells and neutrophils (Mao et al. 2023). Additionally, based on a laser capture microdissection approach, the immune repertoires of intraepithelial T and B cells have been shown to be consistently less diverse and more clonal than those of stromal T and B cells (Romanens et al. 2023b). A comparative analysis of samples from African American women and Caucasian women revealed race-associated differences in regions with immune-rich infiltrates (Bassiouni et al. 2023).

Cancer metabolism has also been investigated in the spatial context of breast cancer, with researchers analyzing primary tumors and paired metastatic lymph nodes showing high levels of oxidative phosphorylation, particularly those located at the tumor leading edge (Liu et al. 2023b).

### Thoracic cancer

The central role of immune checkpoint inhibitors (ICIs) in lung cancer therapy has understandably led researchers to focus their attention largely on the immune system and immune microenvironment. Mechanisms of resistance are now being explored by integrating clinical patient information, such as response to ICI therapy, with ST profiles. For example, enrichment of tumor-associated macrophages (TAMs) in pretreatment samples has been shown to be associated with resistance to immunotherapy treatment in a cohort of 152 patients, with spatial analysis of a subset of these implicating *CD27*, *ITGAM*, and *CCL5* upregulation as drivers (Larroquette et al. 2022). CD68 + macrophages are also enriched with PD1 + and FoxP3 + cells in refractory tumors, while the expression of the IL-2 receptor alpha (CD25) is upregulated in tumor regions of responding patients (Monkman et al. 2023).

Comparative exploration of tumor and adjacent normal lung tissue has also been leveraged to determine the antigenic landscape of so-called “cold” lung cancers, with investigators finding a relatively greater frequency of neoantigens located within HLA-I presentation hotspots in CD3 + CD8 + T-cell-excluded tumors (Kraemer et al. 2023).

Occasionally, patients can present with multiple primary lung cancers, which can be clinically difficult to distinguish from intrapulmonary metastasis. Clinical approaches for differentiating these two phenomena have previously been limited to comparing biopsy samples via histopathological assessment or matching the NGS profiles of each tumor performed as part of standard care. Recently, using scRNA-seq and ST, a newly described population of epithelial cells, termed *CLDN2 +* alveolar type II cells, was shown to be enriched in multiple primary lung cancers (Wang et al. 2023).

Brain metastases from non-small cell lung cancer are a devastating occurrence for patients. The brain has long been known by clinicians as a ‘sanctuary site’ for metastases and is a relatively immune-privileged organ. The functional disruption of immune regulation and fibrosis has now been illustrated using spatial analysis, with T-cell- and antibody-mediated adaptive immune responses being specifically compromised in the brain metastasis microenvironment (Zhang et al. 2022).

### Upper gastrointestinal cancers: gastric, esophageal, pancreatic

Upper gastrointestinal cancers such as gastric cancer and pancreatic cancer are known for being intrinsically resistant to many therapies. The mesenchymal phenotype has been linked to treatment resistance and has been explored with ST in gastric cancer, with the discovery of gastric cancer cells in a partial epithelial–mesenchymal transition state characterized by TGF-β signaling (Jang et al. 2023).

The exact location where a tumor interacts with its surroundings, known as the “tumor-normal interface”, has become an area of great interest with the advent of ST, which is uniquely tailored to this analysis compared to single-cell approaches. Using surgically resected gastric cancer specimens, researchers were able to define an immune cell-dominated tumor-normal interface with distinct immunometabolic alterations, such as disordered arginine and proline metabolism and reprogrammed lipid synthesis (Sun et al. 2023a). ST has also been used in esophageal cancer to elucidate biomarkers of progression from squamous precancerous lesions to carcinoma, with increases in TAGLN2 and decreases in CRNN suggested as candidate indicators for risk of progression (Liu et al. 2023a).

With respect to pancreatic cancer, researchers are investigating scRNA-seq and ST data, and are comparing untreated patient samples with those from patients receiving neoadjuvant therapy and finding a neural-like progenitor malignant cell program that was enriched after neoadjuvant therapy (Hwang et al. 2022). In a similarly designed cohort of treatment-naive and chemotherapy-treated patients, investigators found enrichment of inflammatory cancer-associated fibroblasts (CAFs) that upregulate metallothioneins, coordinated expression of TIGIT in exhausted and regulatory T cells, and individual tumors harboring multiple *KRAS* gene variant hotspots (Cui Zhou et al. 2022). More recently, insights into the immune landscape have been described, including further reports of the exhausted T-cell phenotype and the role of immunosuppressive myeloid cells (Yousuf et al. 2023).

### Lower gastrointestinal cancer: colorectal, liver metastases

Colorectal cancer (CRC) is another tumor where immunotherapy has a limited role, outside of a defined subset of patients who have deficient mismatch repair (MMR) tumors (André et al. 2020). Therefore, immune microenvironment factors have been investigated using ST to identify the mechanisms and therapeutic targets that could improve immunotherapy responsiveness. The colocalization of *FAP +* fibroblasts and *SPP1 +* macrophages in CRC was demonstrated with ST and subsequently analyzed in a patient cohort in which high *FAP* or *SPP1* expression was correlated with decreased benefit from immunotherapy (Qi et al. 2022).

A unique clinical manifestation of colorectal cancer metastasis is its preponderance for isolated spread to the liver, for which surgical resection is often recommended. Comparative analysis of primary and liver metastatic samples has allowed detailed descriptions of their transcriptional differences, which include *MCAM1 +* fibroblast enrichment in liver metastatic tumors (Wang et al. 2022b) as well as the identification of distinct senescent cancer cell subtypes (Garbarino et al. 2023). The invasive edge of liver metastatic tumors has also been characterized by ST, with long-term survivors demonstrating adaptive immune cell populations that are transcriptomically enriched for type II interferon signaling and MHC class II antigen presentation (Wood et al. 2023).

Fascinating insights into the intratumour microbiota have also been made using ST in colorectal cancer, with spatial illustration that bacteria tend to populate niches that are less vascular and highly immunosuppressed, providing evidence of an organized nonrandom distribution of microbiota in these tumors (Galeano Niño et al. 2022). With increasing reports of microbiota detection in other solid tumors, this field of research is likely to accelerate in the coming years.

### Urological cancer: prostate, bladder, kidney

The spatial transcriptome of prostate cancer was investigated as early as 2018 using one of the initial platforms, at the time called ‘*Spatial Transcriptomics’*, which had a lower resolution spot size of 100 μm (Berglund et al. 2018). This initial application of ST provided an atlas-style description of the landscape of prostate tumors with respect to different tissue components. This included stroma, intraepithelial neoplasia, immune cells, and cancer cells. The heterogeneity of metastatic prostate cancer has also been a focus of spatial research, with investigators constructing tissue microarrays of diverse anatomic sites of metastasis. They found that the vast majority of metastases examined had an immune-suppressed microenvironment, as evidenced by a relative absence of inflammatory infiltrates lacking PD-1, PD-L1 and CTLA-4 (Brady et al. 2021). This immune ‘cold’ phenotype was also observed in localized prostate cancer, where ST illustrated the presence of suppressive myeloid populations, exhausted T cells, and high stromal angiogenic activity (Hirz et al. 2023). A unique feature of ST is the ability to infer ligand‒receptor interactions in tissues, with research demonstrating that primary prostate tumors from patients with metastatic disease again exhibit T-cell dysfunction, potentially impaired by nearby regulatory T cells (Salachan et al. 2023).

In bladder cancer, ST has been used to assess differences between primary and recurrent tumor samples, with the recurrent group demonstrating higher degrees of transcriptomic heterogeneity, increased expression of immune response genes and cell adhesion (Lindskrog et al. 2023). Compared with primary tumors, recurrent bladder tumors also exhibit increased cell-to-cell communication between cancer-associated fibroblasts (CAFs) and malignant cells (Shi et al. 2023). Kidney cancer is known to be responsive to immunotherapy, and spatial analysis has revealed that the underlying mechanisms may include myofibroblastic cancer-associated fibroblasts (myCAFs) (Davidson et al. 2023). Elucidating the immune phenotype of high-risk clear cell renal cell carcinoma (ccRCC) with ST has also provided insights into the immunogenic TME of tumors that have abundant exhausted/pro-tumor immune cells and provided clues to mechanisms explaining the infrequent lack of response to immunotherapy, including exhausted immune cells and lack of expression of the *PD-1*, *PD-L1* and *CTLA4* genes (Raghubar et al. 2023).

### Gynecological cancer: ovarian, endometrial

Ovarian high-grade serous carcinoma most commonly presents at an advanced stage; however, research into its earlier precursor, serous tubal intraepithelial carcinoma (STIC), using spatial methods has helped identify increased protein levels of insulin-like growth factor binding protein-2 (IGFBP2) as a mechanism of proliferation (Wang et al. 2022b). High-grade serous carcinoma of the ovary is known to be an aggressive cancer, but despite this, a subset of patients can survive long-term. Spatial analysis of tumor tissue from these patients, compared to that from shorter-term survivors, revealed enrichment of immune cell types infiltrating the tumors of long-term survivors (Ferri-Borgogno et al. 2023).

In endometrial cancer, ST has been integrated into an early-phase clinical trial of a Netrin-1 antibody (NP137), in which biopsy specimens were collected and analyzed to show a treatment-related reduction in epithelial–mesenchymal transition (EMT), allowing simultaneous insights into clinical outcomes and pharmacological mechanisms of action in the spatial context (Cassier et al. 2023).

### Skin cancer: melanoma, cutaneous SCC

Melanoma researchers applied ST methods very early, with initial exploratory studies of melanoma lymph node metastases showing heterogeneity in intra- and intertumoral gene expression (Thrane et al. 2018).

Melanoma brain metastases are a frequent cause of mortality in patients with advanced melanoma. ST combined with scRNA-seq has shown clusters of lymphoid aggregates dominated by plasma cells and, interestingly, has shown spatially differential expression of signatures relating to oxidative phosphorylation and glycolysis. These findings highlight the heterogeneity of these metabolic programs within the metastatic microenvironment (Biermann et al. 2022).

Analogous to cutaneous melanoma, in squamous cell carcinoma of the skin localized disease can be resected and more advanced disease is treated with immunotherapy. Investigators have shown that at the tumor leading edge, CAFs and endothelial transcripts are enriched at the juncture of tumor-specific keratinocytes and adjacent stroma, and the expression of basal tumor genes is increased(Ji et al. 2020).

## Discussion and future directions

The field of spatial transcriptomic cancer research is exploding, and the initial awe-inspiring images that allowed us to visualize the spatial expression of genes in human tissue are truly remarkable breakthroughs. However, we cannot simply empirically accept that “more is better” in regard to extracting information from a biological system or indeed an individual tumor. In its infancy, spatial transcriptomics was applied in a purely investigational or discovery-science approach, as would be expected with any newly developed method.

To realize the transformative possibilities of ST, we need to leverage the unique spatial insights that are being made and bring the findings to patients via a precision oncology approach.

Cancer behavior is now being investigated at the cellular level directly in the tissue context, providing insights into the microenvironment, including different interactions at the tumor leading edge compared to tumor core (Arora et al. 2023; Ji et al. 2020; Y. M. Liu et al. 2023a), which provides clues to the mechanisms of invasion and metastasis. Understanding of the stepwise progression from precancerous to cancerous lesions is also being spatially resolved, which could pave the way forward for early intervention or cancer prevention (Liu et al. 2023a). The central role of the immune microenvironment is now better understood, revealing the various mechanisms that cancer utilizes to cloak itself from recognition, as well as highlighting the exhausted T-cell phenotype and myeloid-derived suppressor populations present in many tumors (Goswami et al. 2023; Ravi et al. 2022a).

The current landscape of literature utilizing ST thus far has been predominantly descriptive in nature but has resulted in some fascinating insights into differing populations, such as long-term survivors compared with short-term survivors (Ferri-Borgogno et al. 2023). While new prognostic factors are welcomed by clinicians, the next step is to harness this knowledge of spatial cancer behavior to inform further research and eventually develop new therapies (Cassier et al. 2023).

The issue of sampling also needs to be kept in mind when putting ST insights into context. Given that multifocal tumors and metastatic deposits are heterogeneous, marrying ST with single-cell and bulk methods, along with direct testing in established research models, will better capture broader tissue heterogeneity and help translate these new findings to patients sooner.

The thoughtful design of clinical trials to include the collection of biospecimens is one factor that may allow ST to take the next step. The future of ST research will be greatly enhanced by a sharper focus on comparative analysis of samples between groups, particularly with respect to their responsiveness to treatment. Cancer treatment selection could be greatly enhanced once we are able to start answering the kind of questions that continue to plague clinicians in the clinic: why did my patient stop responding to immunotherapy? Which combination therapy is best in this situation? Should I treat earlier or later? How did my patient develop resistance to their targeted therapy?

The field is accelerating at a rapid pace, but ultimately, how will spatial transcriptomics help patients with cancer?

As platforms continue to increase their resolution and as costs decrease, this novel method will no doubt have widespread use across future cancer research.

The labor-intensive processing steps and large datasets generated from ST, however, make it unlikely that this kind of analysis could be deployed directly in a clinical pathology laboratory today. However, one potential approach would be to utilize ST on pathology samples where deeper insights are needed from a diagnostic perspective. For example, molecular diagnostics are now being integrated into cancer pathology more frequently to identify targetable pathogenic variants (Dutta et al. 2023). One potential application could be to determine the tumor’s likelihood of being immune-responsive. While this would obviously require validation in a broader population, one could envision a situation in the future where ST could be utilized to identify the subgroup of patients who benefit from immune therapy but do not have the current standard approved biomarkers, such as PD-L1, MSI, or TMB.

Near term benefit for patients from ST research is most likely to be realized through an integrated cycle of research discovery and validation in appropriate settings (Fig. 1). Discoveries made using ST that can be validated in concordant bulk analyses, relevant preclinical models, or translational datasets from clinical trials will be more likely to provide an impact for patients sooner. This process of validation may allow useful surrogates to be identified that have the potential to be integrated into current diagnostic workflows. Emerging methods such as digital pathology analysis could also be enhanced by thoughtful integration of ST data.

Although the timeline for when patients will directly benefit from ST is not entirely clear, it is however clear, that the ST discoveries will undoubtedly be a transformational launching-pad for future precision medicine research, as well as drug discovery, and drug development.

## Data Availability

No datasets were generated or analysed during the current study.
